# A 3D-printed nasopharyngeal swab for COVID-19 diagnostic testing

**DOI:** 10.1186/s41205-020-00076-3

**Published:** 2020-08-15

**Authors:** Jonathan Ford, Todd Goldstein, Sean Trahan, Allison Neuwirth, Kyle Tatoris, Summer Decker

**Affiliations:** 1 Department of Radiology, University of South Florida Morsani College of Medicine, 2 Tampa General Circle STC 6098, Tampa, FL 33606 USA; 2 Northwell Health System, 600 Community Drive Suite 302, Manhasset, NY 11030 USA

**Keywords:** Nasopharyngeal swab, COVID-19, 3D-printing, Point-of-care, Supply chain

## Abstract

The nasopharyngeal swab is a critical component of the COVID-19 testing kit. Supply chain remains greatly impacted by the pandemic. Teams from USF Health Radiology and Northwell Health System developed a 3D-printed stopgap alternative. This descriptive study details the workflow and provides guidance for hospital-based 3D printing labs to leverage the design to make a positive impact on the pandemic. Swab use is also outlined, and the early information regarding clinical use is described, including an ongoing multicenter trial methodology.

## Introduction

The World Health Organization designated COVID-19 a global pandemic on March 11, 2020. In addition to the physical mortality and morbidity on patients, the disruption due to this widespread disease has also effected global supply chain of critically needed medical supplies. Medical supply shortages included ventilators, respirators, masks, face-shields, and various other PPE [1]. 3D printing is an effective stopgap technology for medical devices and additional supplies [2]. There is an international shortage of the synthetic flocked nasopharyngeal (NP) swab which combined with virial transport media in a test tube completes the COVID-19 testing kit [3]. This shortage contributed to deficiencies in testing in the United States.

At this time, the Division of 3D Clinical Applications at the University of South Florida’s (USF) Department of Radiology began looking into the potential for creating a 3D printed alternative for the traditional flocked, NP swab. Using materials that are commonly used in their everyday clinical printing work that were FDA approved for biocompatibility, they were able to come up with a single part design. The printer selection was based on prior FDA approval, available materials and the knowledge that the printer was widely available across the country in many hospitals. Working with colleagues in USF Health Infectious Disease, Otolaryngology and a radiologist, they were able to narrow down the designs to one that would be able to capture a sufficient sample for COVID testing while keeping patient safety and comfort at the forefront. Working with the 3D Design and Innovation lab at Northwell Health, they were able to come up with a design for bench lab testing all in less than a week. Bench lab testing was completed within a couple of days and results immediately demonstrated that the printed swabs were in fact able to detect viral loads and hold those values for 24 h to over 3 days. In light of the bench lab results, a multisite clinical trial was started at Tampa General Hospital (USF Health’s teaching hospital) in order to determine the swab’s clinical efficacy. The clinical trial first tested the traditional swab against the 3D printed swab in known COVID positive patients and later tested in tandem in patients presenting to the emergency rooms. The results showed that the printed swab performed as well as and, in some cases, better than the traditional swab. Early results of the trial from Northwell Health and Tampa General Hospital were presented to the hospital review committees where the 3D printed NP swabs were approved to be the standard of care swab in light of the supply shortage. [Press Release].

As part of the design process, USF Health filed and received a provisional patent [4] on the 3DP NP swab design and concept. The intent was to share the project files for free to appropriate parties like hospitals, clinics, and medical device manufacturers so that any site throughout the world with 3D printing capabilities could use the technology to address these swab shortages. The team specifically approached members of the RSNA 3D Printing Special Interest Group who are trained and approved medical print teams with the designs so that they could help their hospitals. They also joined with FormLabs, the printer manufacturer, in an effort to work with industry for assistance in printing swabs for groups and agencies that were beyond their legal boundaries. Their printers, the Form 2 and Form 3B became the first printers that the NP swabs were printed on nationally.

While USF Health and Northwell Health were the first to test and release the 3DP NP swabs, subsequently numerous other printer companies began working in this area. Several other institutions have tested 3DP alternatives for the swab shortage. For example, Beth Israel Deaconess Medical Center (BIDMC) conducted a robust study examining 160 designs from 48 different materials from 48 different manufacturers, focusing on four designs in a clinical trial [5]. The BIDMC study increases the printer options for manufacturing 3DP swabs, but the machines used are not as ubiquitous as the Form 2 or Form 3B.

To date, there are millions of the USF Health – Northwell Health designed 3D printed NP swab that are actively being produced and used by health care institutions around the US and world.

This paper describes the collaboration between the USF Health and Northwell Health to develop a 3D-printed (3DP) alternative NP swab. Roles and responsibilities of each partner are described. The role of testing is outlined, as is the methodology for a multi-center clinical trial.

## Materials and methods

### Swab design and development

Swabs were printed using Formlabs Form 2 and Form 3B SLA 3D-printers because - in combination with FDA cleared software, they were considered readily available with biocompatible materials and relatively affordable for local deployment.

**Fig. 1:**
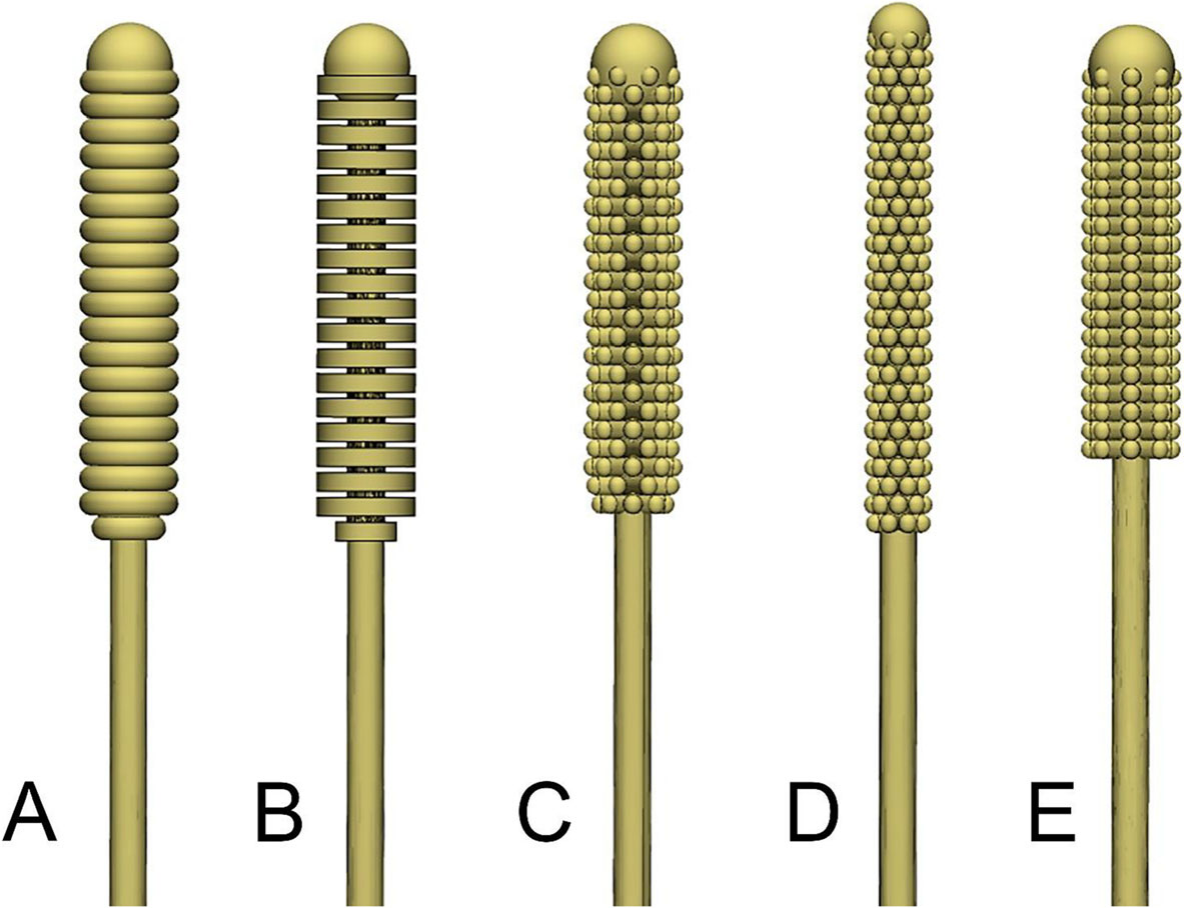
Early alternate 3DP swab designs. Letter C is the current version in use

**Fig. 2:**
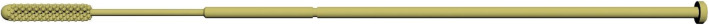
Computer-assisted Drawing (CAD) of NP Swab

Initial prototype computer-aided design (CAD) designs used *Solidworks* (France), *3-matic* (Belgium) or *Fusion 360* (United States). Over twenty tip designs were prototyped, some early examples can be seen in Fig. 1. The goal of tip design was to maximize surface area, sample retention and comfort. Prototypes were narrowed down by clinicians from radiology, infectious disease and otolaryngology. After several design modifications were requested and completed, a final design consisting roughly of a 150 mm in total length with a 70 mm breakpoint. The tip length is 15 mm long, with a diameter of 3.85 mm. The tip has a rounded nose for patient comfort. The neck is 1.5 mm in diameter and the shaft is 2.45 mm in diameter. The base is 1.75 mm long with a 5 mm diameter (Fig. 2). As each 3DP swab utilizes approximately 0.76 ml of resin to print (when combined with post-processing materials and potential waste), each swab costs approximately $0.25 USD to print. “Tip C” is the current tip configuration that is in use presently. “Tip D” is an experimental swab designed for smaller nasal passages made at the request of our clinicians.

Bench lab testing of the final swab designs was conducted to ensure the geometries picked up enough of a sample to allow for viral testing. Swab materials were incubated for up to 3 days to ensure the printer resin would not interfere with downstream viral testing. As viral transport media (VTM) is also potentially in short supply, additional testing was used utilizing in house mixing of VTM using the World Health Organization recipe [6].

### Current algorithm for swab printing

**Fig. 3:**
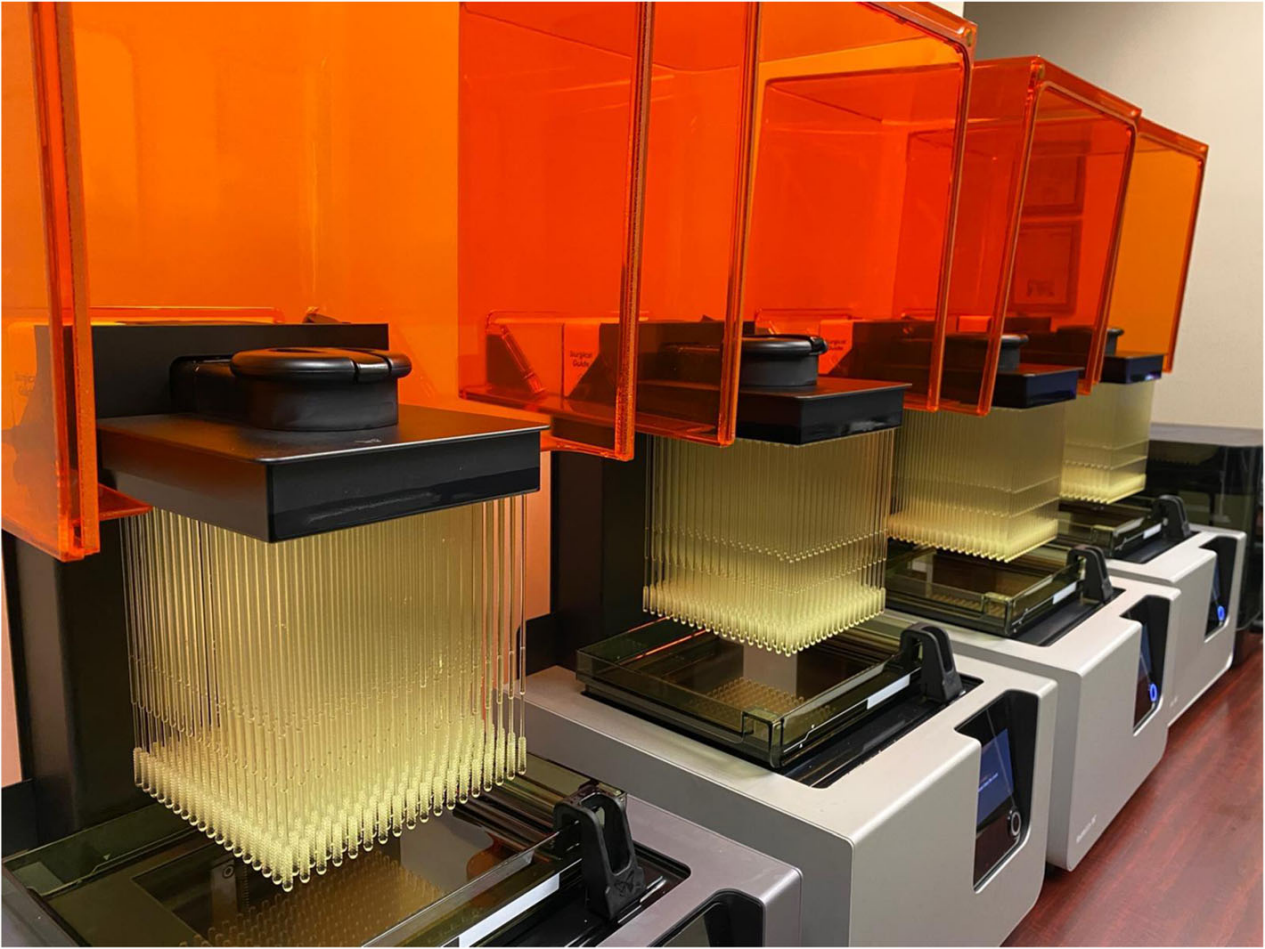
Four batches of 324 3DP NP swans ready for post-processing

The STL of the 3DP swab was then arrayed for 3D-printing using the Preform software. A balance of printing the most swabs and limiting the contact points to avoid any potential issues determined that 324–380 swabs could be safely printed per batch (Fig. 3). Swabs are printed at a layer thickness of 100 μm (the fastest setting) using Surgical Guide version 1 resin, with the base directly in contact with the platform with no rafts and no supports.

**Fig. 4:**
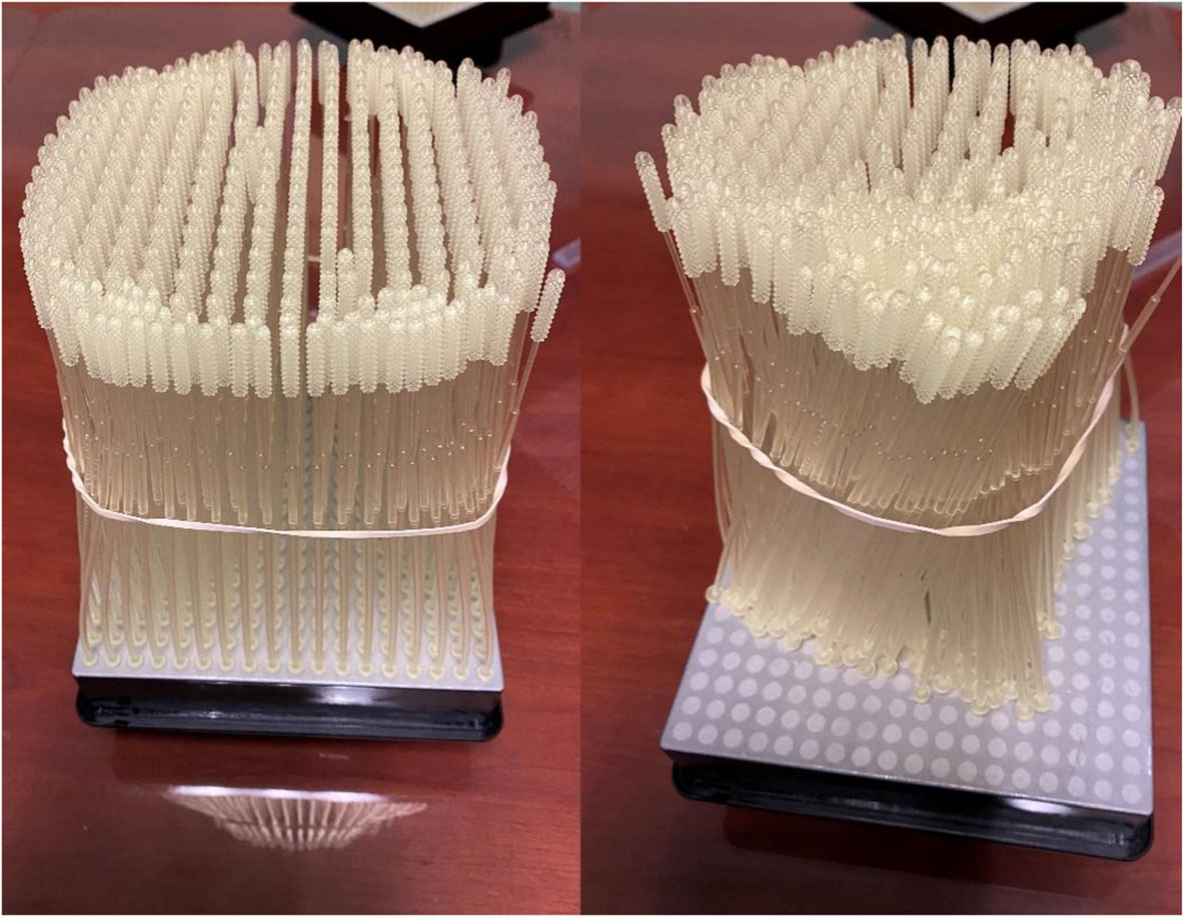
Example of a loose rubber-band (elastic) to assist in swab removal from platform

3DP swabs are rinsed using a Form Wash for 20 min in 99% isopropyl alcohol. This washing process is performed while the swabs are still attached to the build plate. Manual washing may be possible using the Finishing Kit, however swabs would need to be removed from the platform first. Once the wash is complete, the prints are allowed to airdry for at least 30 min. Afterwards the swabs are gently scraped off the build platform. The placing of a loose rubber band around the printed swabs prior to scraping them from the platform assisted in overall organization (Fig. 4).

**Fig. 5:**
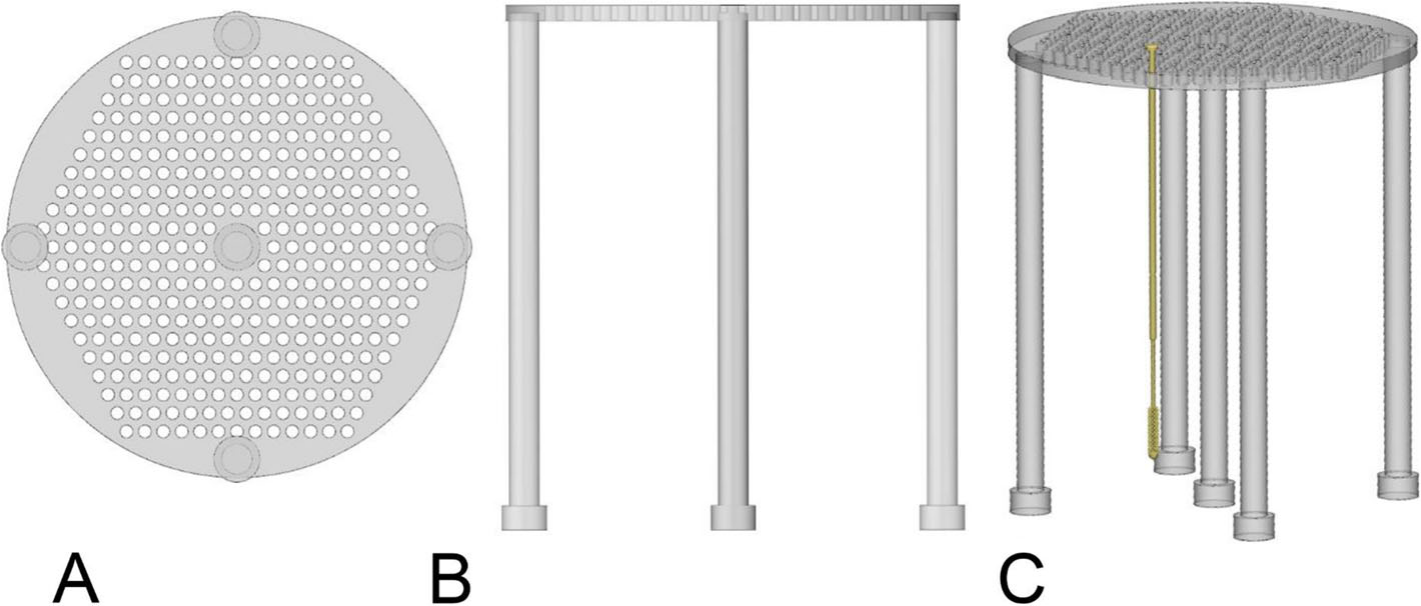
**a**: Top, **b**: Side and **c**: Three-Quarter View of Curing Rack

The swabs are then suspended from the base (with the tip pointing down) in a curing rack and placed in the Form Cure for 60 °C for 30 min (Form 2) or 70 °C for 30 min (Form 3B). An example of a curing rack can be seen in Fig. 5. The inverted suspension of the NP swabs by a rack during curing removed issues with bent necks or shafts. It is recommended that the curing rack not be overcrowded to ensure that enough airflow and UV exposure occurs for each swab. Once the curing is complete, 3DP swabs are placed in steam sterilization pouches, and prepared for sterilization. Pre-vacuum steam sterilization cycle set at 132 °C/270 °F with a 4 min sterilization phase and 30 min dry is an appropriate sterilization cycle. Additionally, swabs may be treated in Prolystica 2X Enzymatic Presoak and Cleaner prior to pouching.

**Fig. 6:**
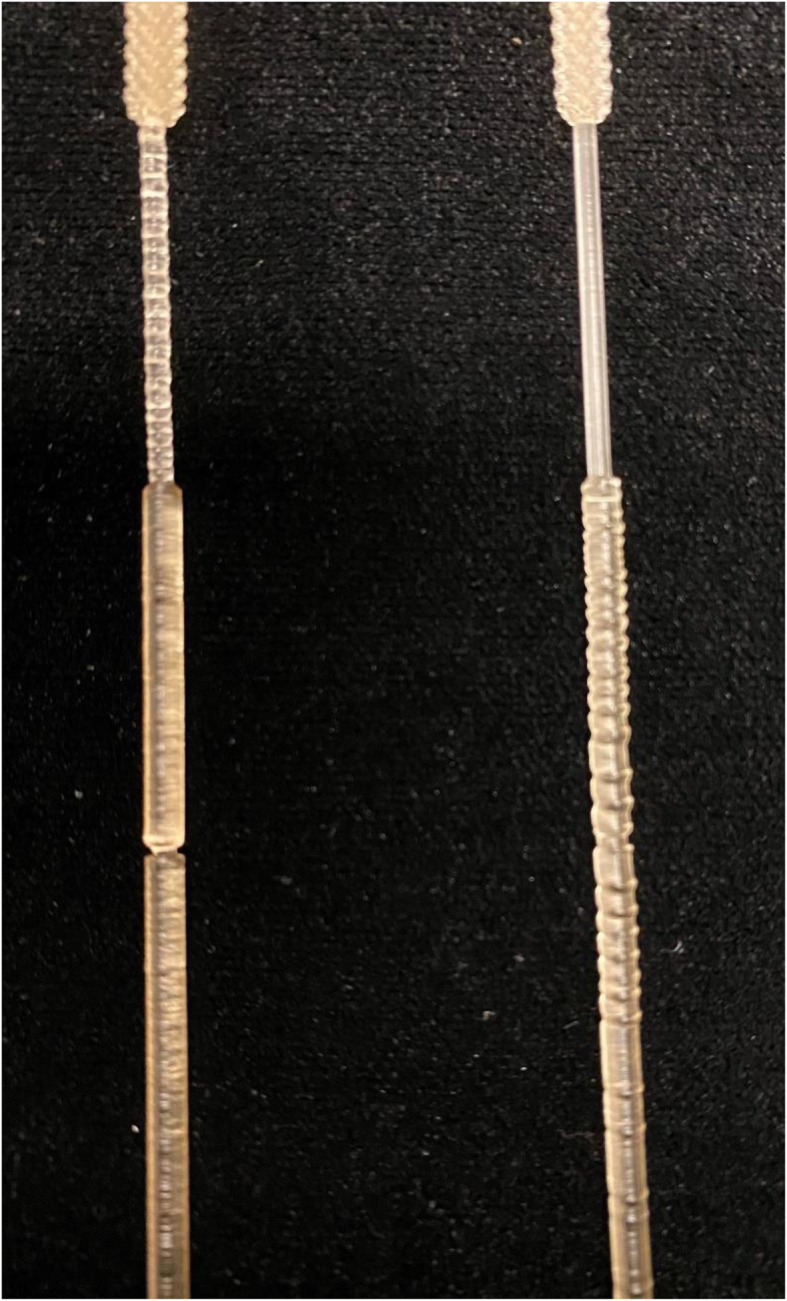
Example of “jittery” failed swab

Several differences were also noted between the Form 2 and Form 3B. Form 2 s printed at 15–16 h per batch. Form 3Bs initially printed between 26 and 36 per batch. However, firmware updates and software settings have lowered print times to 10–11 h. Form 3Bs also have greater reject rates with some swabs failing to adhere to the build platform as well as higher rates of print inconsistencies with a “jittery” effect noted (Fig. 6).

There are several considerations for those planning to utilize this workflow. The Surgical Guide resin is a photo-reactive polymer. Once the swabs have been sterilized, they should be kept out of direct light prior to use to prevent them from becoming more brittle over time. Storing them in a dark location prior to use would increase shelf-life. Additionally, isopropyl alcohol of 99% is critical to the washing cycle and due to high market demands, be sure it can be acquired readily.

## Discussion

### Implementation of swab in practice

At USF Health, 3DP swabs are produced and post-processed (washed and cured) prior to being sent for sterilization. The swabs are individual packaged and autoclaved then are bundled with the test tube and VTM to complete the COVID-19 testing kit. These kits are then provided to the testing sites for use. Once the samples are collected, the swabs are broken off into a test tube containing VTM and then PCR analysis is run in our diagnostic laboratories.

Several meetings have been held with the US Food and Drug Administration (FDA) with respect to the usage of 3DP swabs. The 3DP swabs have been declared a Class 1 exempt medical device and is subject to the least amount of regulatory control. Potential users should closely follow any relevant FDA, NIH, and VA announcements and advisories in regard its use and testing capabilities. There are restrictions on who can legally print and provide 3DP swabs. Hospitals and health care facilities can produce their own swabs and provide them for their own in-house use and to their affiliate sites. Licensed medical device manufacturers may also produce 3DP nasopharyngeal swabs. It is advised that any parties seek legal counsel to ensure they are within their legal bounds prior to attempting to print the swabs. At the time of submission for publication, USF Health and Northwell Health combined have produced several hundred-thousand swabs. Across the globe in a variety of institutions and countries the current estimate is that 25 million swabs have been produced.

At the USF Health and Northwell Health facilities, 3DP swabs are being assembled as part of a test kit. Internal laboratories at each respective hospital are collecting patient samples and testing them in-house. As of the time of this publication, two of the leading medical testing services, LabCorp and Quest Diagnostics, are waiting for an official statement from the FDA prior to accepting the 3DP swab.

### Clinical effectiveness

USF Health/Tampa General Hospital (TGH) started a multisite clinical trial that is currently underway. This trial is testing the 3DP swab against the current synthetic version. USF Health/TGH as well as Northwell Health are conducting head-to-head trials utilizing each swab on the same patient in alternate nostrils. Study participants include any patients suspected of COVID-19 or current patients in the hospital with a known COVID-19 diagnosis. The initial results at both USF Health/TGH and Northwell Health show a 94% concurrence with no statistical differences between the threshold cycle (Ct) values when comparing the 3DP swab against the synthetic swab. Both hospitals have concluded that the 3DP swab is a viable alternative to the synthetic swab and are now using them as their standard of care swab. Several other sites participating in the clinical trial are in different stages of completion. A full publication and study will be published upon the conclusion of the trial.

### Impact

The response and impact of the USF Health – Northwell Health 3DP NP swab has been tremendous. While the clinical trials are ongoing at many sites, numerous states around the US including New York, Florida, Massachusetts, Kansas, Oklahoma, Virginia, and Ohio all have been using the design at the state level with Ohio having over 1 million alone. Additionally, all branches of the US military have been working with the USF Health and FormLabs teams to develop the design for COVID testing within the military. The swab has also expanded internationally to Canada, Colombia, Brazil, Japan, Switzerland, Argentina, Malaysia, Georgia, the Philippines, and the United Kingdom. The invention has been featured in the media, like *Forbes* and the *Wall Street Journal*, nationally and internationally as an example of the power of medical 3D printing and quick innovation for clinical solutions.

## Conclusion

3D printed nasopharyngeal swabs provide a cost-efficient and fast alternative to the standard NP swabs used for COVID-19 testing kits. As members of the Radiological Society of North America’s 3D Printing Special Interest group [7], this project was initiated and developed through collaborations established in the 3D SIG.

Point-of-care or in hospital 3D printing is uniquely positioned to understand the immediate needs of a hospital and act on them quickly. By working together between hospitals and industry during the pandemic, valuable time was saved to address the shortage needs in our respective hospitals. The in-house production of these 3DP swabs alleviated this major supply chain hurdle and directly increased testing capabilities at USF Health, Tampa General Hospital and Northwell Health System.

Today there are millions of 3D printed nasal swabs around the world demonstrating the field of 3D printing’s ability to address the supply shortages of critical need resources used for COVID testing.

## Data Availability

Print files available upon request at: 3dclinicalapplications@usf.edu

## Abbreviations

3DP: 3D-Printing, 3D-printed
NP: Nasopharyngeal
POC: point-of-care
